# Lead and IQ in Children: Lanphear et al. Respond

**Published:** 2006-02

**Authors:** Bruce P. Lanphear, Richard Hornung, Jane Khoury, Kimberly Yolton, Kim N. Dietrich

**Affiliations:** Departments of Pediatrics and of Environmental Health, Cincinnati Children’s Hospital Medical Center, Cincinnati, Ohio, E-mail: bruce.lanphear@chmcc.org

As described in our original article (Lanphear et al. 2005), we decided *a priori* to focus our analyses on the blood lead (BPb) variable that had the strongest association with IQ (intelligence quotient) scores. This decision was made to limit the number of analyses and to minimize problems with multiple comparisons. There was a clear consensus among the co-investigators—which originally included Ernhart—to use this strategy. Because concurrent BPb concentration was the strongest predictor of intellectual functioning, we focused most of our analyses on this variable.

There are now several studies indicating that concurrent or lifetime average BPb concentration are better predictors of children’s IQ scores than measures taken in early childhood (Baghurst et al. 1992; Canfield et al. 2003; Chen et al. 2005; Dietrich et al. 1993; Tong et al. 1996; Wasserman et al. 2003). Thus, existing evidence indicates that interpretation of this literature should rely on concurrent or lifetime measures of BPb concentration.

Ernhart is concerned that we found no significant association of IQ and three of the four indices of lead exposure at peak BPb levels < 10 μg/dL or < 7.5 μg/dL (Lanphear et al. 2005). In addition to a significant inverse association of concurrent BPb concentration and IQ score for children with maximal BPb levels < 7.5 μg/dL, we found a consistent inverse association for lifetime average BPb concentration (β = −3.13, *p* = 0.054). As we reported, the relationship of peak BPb concentration and early childhood BPb concentration was not as predictive of children’s IQ scores.

Ernhart is particularly concerned about our analyses for children with “very low” lead exposure (Lanphear et al. 2005). The results of our parsimonious analysis for children who had maximal BPb concentrations < 10 μg/dL and < 7.5 μg/dL were entirely consistent with the fully adjusted model. When we included all of the additional covariates, concurrent BPb concentration changed by < 5%, and it remained statistically significant (β = −2.99, *p* = 0.019) for the children with maximal BPb levels < 7.5 μg/dL. When we further restricted the analysis to U.S. cohorts and introduced race as a covariate, race was clearly not a significant factor, and the pattern remained similar. These secondary analyses support our original conclusion that there is an inverse relationship of lead exposure and intellectual function, with greater decrements at lower BPb concentrations (Lanphear et al. 2005).

We agree that using an early measure of the HOME (Home Observation for Measurement of the Environment) inventory (Caldwell and Bradley 1984) in the Rochester cohort was a potential limitation. Still, when we excluded the Rochester cohort from the pooled analysis, the coefficient changed by < 3% and remained statistically significant (Lanphear et al. 2005). Thus, this limitation did not alter the conclusions of the study.

Ernhart is critical about the “peculiar” increase in sample size and shifts in demographic variables in the Rochester study. Although some families became “too busy” to participate when their children were toddlers, we routinely invited them to participate in subsequent visits. A larger number of families were willing to return for an evaluation as their children aged.

We conducted a secondary analysis of studies that included prenatal BPb concentration. Contrary to Ernhart’s comment, prenatal lead exposure was not significantly associated with children’s IQ scores in adjusted analyses (Lanphear et al. 2005). We concluded that “prevention of lead exposure must occur before pregnancy or a child’s birth” (Lanphear et al. 2005) because children are particularly vulnerable to lead intake and absorption during the first 2–3 years of life (Clark et al. 1985; Lanphear et al. 2002; Ziegler et al. 1978).

Ernhart argues that her request for further information about the 6-year data from the Rochester study “was denied.” The earlier measures of intellectual function in the Rochester children (i.e., at 3 and 5 years of age) were measured using the Stanford Binet test (Canfield et al. 2003). The IQ test at 6 years of age, which was measured using the Wechsler Preschool and Primary Scales of Intelligence, was done specifically for the pooled analysis. We believed that it was in the best interest of public health to confirm the findings of the original Rochester study with the larger pooled analysis rather than await publication of the follow-up 6-year IQ tests.

Numerous studies have found evidence for adverse consequences of childhood lead exposure at BPb levels < 10 μg/dL (Bellinger and Needleman 2003; Chiodo et al. 2004; Fulton et al. 1987; Lanphear 2000; Sood et al. 2001; Walkowiak et al. 1998; Wasserman et al. 2000). These studies provide sufficient evidence that childhood lead exposure should be reduced even more by banning all nonessential uses of lead and further reducing the allowable levels of lead in air emissions, house dust, soil, water, and consumer products.
